# Environmental factors prevail over dispersal constraints in determining the distribution and assembly of Trichoptera species in mountain lakes

**DOI:** 10.1002/ece3.1522

**Published:** 2015-06-01

**Authors:** Guillermo de Mendoza, Marc Ventura, Jordi Catalan

**Affiliations:** 1 Centre for Advanced Studies of Blanes (CEAB), Spanish National Research Council (CSIC)Accés a la Cala St. Francesc 14, E-17300, Blanes, Catalonia, Spain; 2 Centre for Ecological Research and Forestry Applications (CREAF)Campus UAB, Edifici C, E-08193, Cerdanyola del Vallés, Catalonia, Spain

**Keywords:** Alpine lakes, altitudinal gradient, macroinvertebrates, niche segregation, spatial autocorrelation, spatial scale

## Abstract

Aiming to elucidate whether large-scale dispersal factors or environmental species sorting prevail in determining patterns of Trichoptera species composition in mountain lakes, we analyzed the distribution and assembly of the most common Trichoptera (*Plectrocnemia laetabilis*, *Polycentropus flavomaculatus*, *Drusus rectus*, *Annitella pyrenaea,* and *Mystacides azurea*) in the mountain lakes of the Pyrenees (Spain, France, Andorra) based on a survey of 82 lakes covering the geographical and environmental extremes of the lake district. Spatial autocorrelation in species composition was determined using Moran’s eigenvector maps (MEM). Redundancy analysis (RDA) was applied to explore the influence of MEM variables and in-lake, and catchment environmental variables on Trichoptera assemblages. Variance partitioning analysis (partial RDA) revealed the fraction of species composition variation that could be attributed uniquely to either environmental variability or MEM variables. Finally, the distribution of individual species was analyzed in relation to specific environmental factors using binomial generalized linear models (GLM). Trichoptera assemblages showed spatial structure. However, the most relevant environmental variables in the RDA (i.e., temperature and woody vegetation in-lake catchments) were also related with spatial variables (i.e., altitude and longitude). Partial RDA revealed that the fraction of variation in species composition that was uniquely explained by environmental variability was larger than that uniquely explained by MEM variables. GLM results showed that the distribution of species with longitudinal bias is related to specific environmental factors with geographical trend. The environmental dependence found agrees with the particular traits of each species. We conclude that Trichoptera species distribution and composition in the lakes of the Pyrenees are governed predominantly by local environmental factors, rather than by dispersal constraints. For boreal lakes, with similar environmental conditions, a strong role of dispersal capacity has been suggested. Further investigation should address the role of spatial scaling, namely absolute geographical distances constraining dispersal and steepness of environmental gradients at short distances.

## Introduction

Analyzing the relative importance of local environmental factors with respect to large-scale dispersal restrictions is fundamental for understanding species distributions and community composition at regional scale (e.g., Shurin 2000; Chase 2003; Soininen et al. 2007). Under a scenario purely driven by dispersal, the assembly of communities depends on the stochastic nature of the colonization and the assemblages are prone to multiple stable states driven by priority effects, whereby early colonizers exert a strong influence on the subsequent settlement of new species, eventually affecting community assemblages (Louette et al. 2008; Chase 2010). Thus, the similarity among communities tends to depend on the geographical distance between them. Alternatively, when local environmental factors exert a strong filter for colonizers, the similarity between communities depends less on geographical distance than on the resemblance of key environmental conditions among sites (Chase 2007).

Whether dispersal or environmental constraints prevail depends on the characteristics of both the organisms and the spatial scale considered, as shown for stream insect communities (Bonada et al. 2012; Landeiro et al. 2012; Heino 2013; Heino and Peckarsky 2014). In the mountains, however, it is also necessary to consider the altitudinal gradient, which encompasses stronger environmental changes across shorter spatial distances (particularly with regard to temperature) than it would be the case of an extensive landscape without changes in elevation. Here, we analyzed the aquatic community structure and species distribution of Trichoptera (Arthropoda: Insecta), from a survey of 82 lakes in the Pyrenees ranging in altitude from 1620 to 2990 m a.s.l. (de Mendoza and Catalan 2010) by considering in-lake and catchment environmental factors, and the structure of the spatial autocorrelation shown by the species assemblages.

Trichoptera, commonly called “caddisflies”, are among the most diversified groups of aquatic insects, comprising more than forty families (Holzenthal et al. 2007) and covering a wide range of functional larval types (Cummins 1973; Wissinger et al. 1996, 2003; Tachet et al. 2010). There is higher caddisfly diversity in running (lotic) waters than in lentic systems (e.g., lakes), in which not all families are present (Mackay and Wiggins 1979). This is attributed to the probable origin of Trichoptera in cool running waters (Ross 1967; Wiggins 2004), and the diversification according to the high hydrodynamic heterogeneity existing in these systems (Statzner and Higler 1986). Hydrodynamics becomes an adaptive factor both, directly, to prevent uncontrolled drift (Wallace and Anderson 1996) and, indirectly, because the materials available for case building are tightly related to the hydraulic conditions (Hynes 1970); for instance, mineral grains of adequate size can be a limited resource for those Trichoptera larvae using grains to build their cases (Statzner 2011). Therefore, the caddisfly species found in lakes tend to be those inhabiting slow-current zones in streams.

There are few studies focusing on the factors determining the distribution of Trichoptera species in lentic systems, yet Trichoptera are indeed a common group of macroinvertebrates in high-mountain and boreal lakes (Knapp et al. 2001; Raddum and Fjellheim 2002; Boggero and Lencioni 2006; Krno et al. 2006; Wissinger et al. 2006). In lakes, there is less hydrodynamic heterogeneity than in rivers and, accordingly, it could be expected that regional dispersal constraints such as geomorphological barriers across valleys could be more relevant than environmental filters in determining the caddisflies distribution. In agreement with this hypothesis, the study of 99 boreal lakes in central Sweden by Hoffsten (2004) suggested that dispersal processes are strong determinants of the Trichoptera species distribution in these systems and one species, *Agrypnia obsoleta* (Hagen), with high capability for dispersal, showed a very high occupancy. Mountain lake districts provide similar environmental conditions as those of the boreal sites sampled by Hoffsten (2004) but in a rather different spatial setting (Söderberg and Norrgrann 2001; Catalan et al. 2009). The environmental contrast at short-spatial scales is stronger in high mountains than in boreal areas owing to the altitudinal gradient. Here, we aim to analyze whether this scaling feature may enhance the influence of environmental filtering in the species distribution.

## Materials and Methods

### Lake selection and sampling

**Figure 1:**
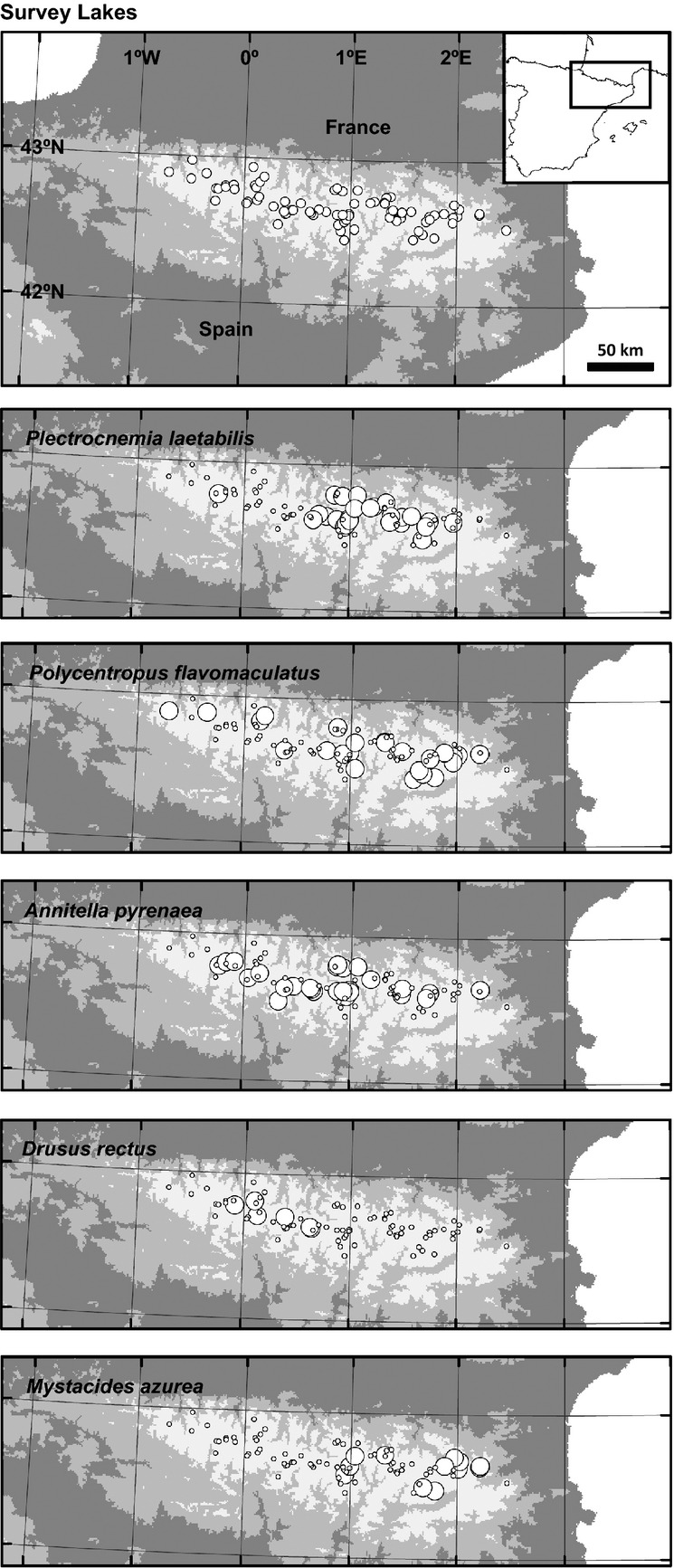
Geographical distribution of the lakes surveyed and the five most frequent Trichoptera species. Large circles indicate the respective species presence. Appendix S1 includes the detailed distributions of all the taxa found.

We selected 82 representative high-mountain lakes ranging from 1620 to 2990 m a.s.l. (Fig.1) based on the altitude and lithology of lake catchments (de Mendoza and Catalan 2010) as these two factors, respectively, determine most of the physical (Thompson et al. 2009) and chemical variability in mountain lakes (Catalan et al. 1993; Camarero et al. 2009). Lakes at geographical extremes were also included in order to consider the boundaries of the lake district area, and lakes of different size were also representatively chosen within each altitude–lithology category when possible. Sampling was performed during the summer of 2000 in the littoral zone of lakes (ca. 80 cm depth), which was assumed to be deep enough to avoid the potential damage of benthos caused by freezing periods, but still shallow enough to ensure the highest number of Trichoptera species to be found as shown by other studies (Capblancq and Laville 1983; Rieradevall and Prat 2000). The kick-sampling technique of Frost et al. (1971) was used with a pond net of 100 *μ*m mesh size (250 *μ*m mesh-size sieve eventually used in the laboratory), at five 1-m^2^ sampling points per lake and during 1 min in each. Sampling points were selected so as to cover the different habitat types in each lake, and the number of sampling points assigned to a habitat type was weighted according to the habitat proportion in the whole littoral zone, which was assessed by a previous in situ exploration of the entire lake perimeter by several observers. The type of habitats sampled comprised presence/absence of macrophytes as well as different bottom substrates (i.e., fine substrates, gravel, stones, and rocks). Large stones were turned over and brushed in the net when they were present.

### Taxonomic determination

For taxonomic determination, general references of the Palearctic region were used, complemented with reference to more specialized taxonomic papers on larvae and mature pupae of Trichoptera (see Appendix S1 in Supporting Information). Not all individuals could be determined to the species level, and some words of caution are necessary concerning *Plectrocnemia*, *Annitella,* and *Drusus* species assignments, as indicated in Appendix S1. The detailed distribution of all Trichoptera taxa found in the lakes studied is shown in Appendix S1.

### Environmental variables

Environmental variables were measured or determined in the field, or from water samples taken at the time of the Trichoptera sampling, and complemented with auxiliary information from other sources (see below). We grouped the environmental variables that potentially could explain the species assemblage into two groups, namely in-lake and catchment variables (descriptive statistics for all variables are given in Appendix S2).

The in-lake group included descriptors of the physical and chemical environment, general lake trophic status, littoral substrate, and some biotic conditions (Catalan et al. 2009), namely lake area; lake depth; conductivity; pH; total nitrogen (TN); total phosphorus (TP); dissolved organic carbon (DOC); dissolved silica; ammonium; calcium; magnesium; sodium; potassium; sulfate; nitrate; chloride; acid neutralizing capacity (ANC); surface water temperature; organic matter in deep sediment, estimated as loss on ignition (LOI); chlorophyll-*a* (Chl-*a*); bacteria as biomass in plankton samples; granulometry of the substrate as mean relative abundance of “rocks”, “stones”, “gravel”, and “fine substrate” (estimated by an in situ exploration of the lake littoral zone by several observers independently); macrophyte dominance; and fish occurrence classified as “Salmonidae” and “*Phoxinus*”, to refer to any *Salmo*, *Salvelinus* or *Oncorhynchus,* and *Phoxinus* species, respectively. Samples for all variables were collected (temperature directly measured) at the outlet, except for LOI, Chl-*a,* and bacteria. For Chl-*a* and bacteria samples were collected at the depth of 1.5-fold the Secchi disk depth, corresponding to the deep chlorophyll-*a* maximum (Catalan et al. 2002). The analytical methods used are described in Ventura et al. (2000), with the exception of LOI, determined according to Heiri et al. (2001), and bacteria biomass, determined following Straškrabová et al. (1999). The classification of fish occurrence into the two nominal categories (“Salmonidae” and “*Phoxinus*”) was obtained from Miró and Ventura (2013, 2015). Lake and catchment areas were determined using orthophotomaps and geographical information systems, and lake depth was measured in the field with a portable echo sounder.

The catchment variables included landscape units considered as nonoverlapping vegetation or geomorphological elements (“woody vegetation”, “meadows”, “rocky meadows”, “peat bog”, “scree”, “bare rocks”, “glaciers”, and “glacial deposits”); bedrock relative composition (“metamorphic rocks”, “plutonic rocks”, “detrital rocks”, and “carbonate rocks”); and catchment area. The relative dominance of these units was estimated by the in situ exploration of lake catchments by several observers, cartographic information, and satellite imagery (Casals-Carrasco et al. 2009).

### Numerical methods

Only species present in more than five lakes were considered for statistical analyses. As a first exploratory step, the potential bias of species in their geographical distribution was explored by analyzing segregation patterns of these species through a series of Student’s *t*-tests (equal variances not assumed) comparing the mean altitude, longitude, or latitude values between lakes with, and without, a given species (Zar 1984).

The spatial autocorrelation present in the species assemblages in a two-dimensional space (i.e., longitude and latitude) was analyzed by means of Moran’s eigenvector maps (MEMs) (Dray et al. 2006, 2012; Borcard et al. 2011) after estimating the most likely connectivity matrix operating between lakes using the packages “SoDA” (Chambers 2013) and “spacemakeR” (Dray 2013), available in R software (R Core Team, 2013) (see Appendix S3). MEMs represent patterns of spatial autocorrelation in the species distributions and specifically are the result of the spectral decomposition of the spatial relationships among the samples as defined by the Moran’s *I* statistic (Dray et al. 2012). Positive MEM variables, indicating positive spatial autocorrelation, were used to explain species assemblages using redundancy analysis (RDA) (Legendre and Legendre 1998; Borcard et al. 2011), considering only lakes where at least one of the common species was found (*n* = 60). RDA is suitable for this purpose after appropriate transformation of raw species data to obtain a Hellinger distance ordination (Legendre and Gallagher 2001). MEM variables were selected in RDA by forward selection (*P* < 0.05, 9999 Monte Carlo permutations) in which the double-stopping criterion of Blanchet et al. (2008) was applied. The species composition variance explained was always considered in terms of adjusted *R*^2^ values (Peres-Neto et al. 2006). RDAs were performed with the R packages “vegan” (Oksanen et al. 2013) and “packfor” (Dray et al. 2013) (further details in Appendix S3).

The relationship between the species assemblages and the environment was also analyzed with RDA following the same procedure, with either in-lake or catchment variables. This RDA also allowed an exploration of individual species–environment relationships. As with MEM variables, the original pool of explanatory variables was reduced by forward selection of variables within each group (in-lake and catchment). Environmental variables departing from normality in a Kolmogorov–Smirnov (KS) goodness-of-fit test (Zar 1984) were previously log-transformed. Specifically, the only variables not log-transformed were the habitat variables and pH. For catchment variables, the log-transformation was performed as log (*x* + 1), in order to avoid zeros which do not permit logarithmic transformation; for some in-lake variables, the zeros and negative values (ANC) were transformed into a very small positive number, one order of magnitude below the lowest positive value measured (i.e., 0.001 for K^+^, 0.01 for DOC and 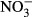, and 0.1 for ANC).

Variance partitioning of the species composition between environment and spatial structure was performed by partial RDA (Legendre and Legendre 1998; Oksanen et al. 2013). The overall linear trend (corresponding to longitude) present in the data was incorporated explicitly in partial RDA following Borcard et al. (2011) in addition to MEM, in-lake, and catchment variables. In order to explore unconstrained relationships between specific environmental factors and MEMs, the Pearson product-moment correlation coefficient *r* was used to evaluate pair-wise relationships between the two types of variables.

Finally, the most influential environmental factor on the distribution (presence/absence) of each species was determined by generalized linear models (GLMs) (Zuur et al. 2007) using the same lake set as in the previous RDAs (*n* = 60). All variables at our disposal were considered. We performed binomial logistic GLMs in R (R Core Team, 2013) using one environmental variable at a time, and the most adequate model was defined as the one with the lowest AIC value (Akaike 1973). Nevertheless, all the models with AIC values that were at most two units higher than the lowest AIC value were recorded following Burnham and Anderson (2002). The relevance of each variable for each species was defined as the percentage of null deviance explained by the model with that variable, and its significance was evaluated with chi-squared tests on a deviance table after checking for overdispersion (Zuur et al. 2007). The probability of occurrence of each species as a function of the most adequate variable was plotted using binomial logistic regression (R Core Team, 2013).

## Results

### Trichoptera in the lakes of the Pyrenees

We found 10 Trichoptera taxa (Appendix S1), five of which were considered for statistical analyses as they were present in more than five lakes: *Plectrocnemia laetabilis* McLachlan and *Polycentropus flavomaculatus* (Pictet) (Polycentropodidae); *Annitella pyrenaea* (Navás) and *Drusus rectus* McLachlan (Limnephilidae); and *Mystacides azurea* (Linnaeus) (Leptoceridae). Geographical patterns were observed in the distribution of each species except for *A. pyrenaea*. The species *P. laetabilis*, *P. flavomaculatus, and M. azurea* were mostly confined to eastern and *D. rectus* to western Pyrenees (Fig.1, Table1). Both *P. flavomaculatus* and *M. azurea* showed a negative altitudinal bias and *D. rectus* a positive bias. Finally, *M. azurea* showed an apparent southward latitudinal bias as eastern lakes are also located further south.

**Table 1:** Incidence (frequency of occurrence) and abundance of the five most common Trichoptera found in the lake survey (*n* = 82), together with their altitudinal, longitudinal, and latitudinal ranges

	*Plectrocnemia laetabilis*	*Polycentropus flavomaculatus*	*Annitella pyrenaea*	*Drusus rectus*	*Mystacides azurea*	All lakes
Incidence	25	24	27	6	12	60
Abundance total	341	185	246	26	95	893
Altitude (m a.s.l.)						
Altitude minimum	1929	1875	1804	2537	1920	1620
Altitude maximum	2531	2550	2740	2740	2440	2990
Altitude mean	2303	2224	2316	2626	2124	2302
*P*-value	0.980	**0.041 (−)**	0.744	**<0.001 (+)**	**0.004 (−)**	–
Longitude (°E)						
Longitude minimum	−0.242	−0.706	−0.242	−0.088	0.951	−0.706
Longitude maximum	1.967	2.211	2.214	0.638	2.214	2.463
Longitude mean	1.149	1.165	0.798	0.298	1.675	0.890
*P*-value	**0.012 (+)**	**0.043 (+)**	0.394	**0.001 (−)**	**<0.001 (+)**	–
Latitude (°N)						
Latitude minimum	42.498	42.458	42.545	42.630	42.474	42.451
Latitude maximum	42.794	42.884	42.808	42.810	42.712	42.968
Latitude mean	42.659	42.657	42.676	42.711	42.626	42.676
*P*-value	0.263	0.348	0.985	0.273	**0.036 (−)**	–

*P*-values refer to two-tailed Student’s *t*-tests (equal variances not assumed) comparing mean values of altitude between lakes with, and without, a given taxon (the geographical bias in distributions is shown as a sign in brackets). Significant *P*-values (*P* < 0.05) are shown in boldface. The detailed distribution of all the Trichoptera taxa found is shown in Appendix S1.

### Spatial autocorrelation

**Figure 2:**
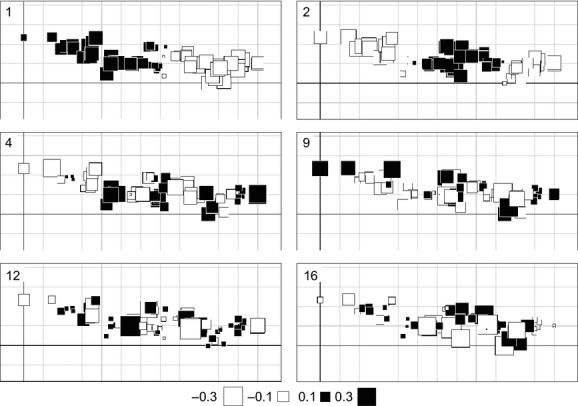
Moran’s eigenvector maps (MEM) selected as significant (*P* < 0.05 after 9999 Monte Carlo permutations) in explaining the spatial autocorrelation of Trichoptera distributional data with redundancy analysis (RDA). The color and size of square symbols represent site scores for each MEM, as indicated in the legend below graphs. Appendix S3 includes the estimation of the lake connectivity matrix for MEM analysis.

**Figure 3:**
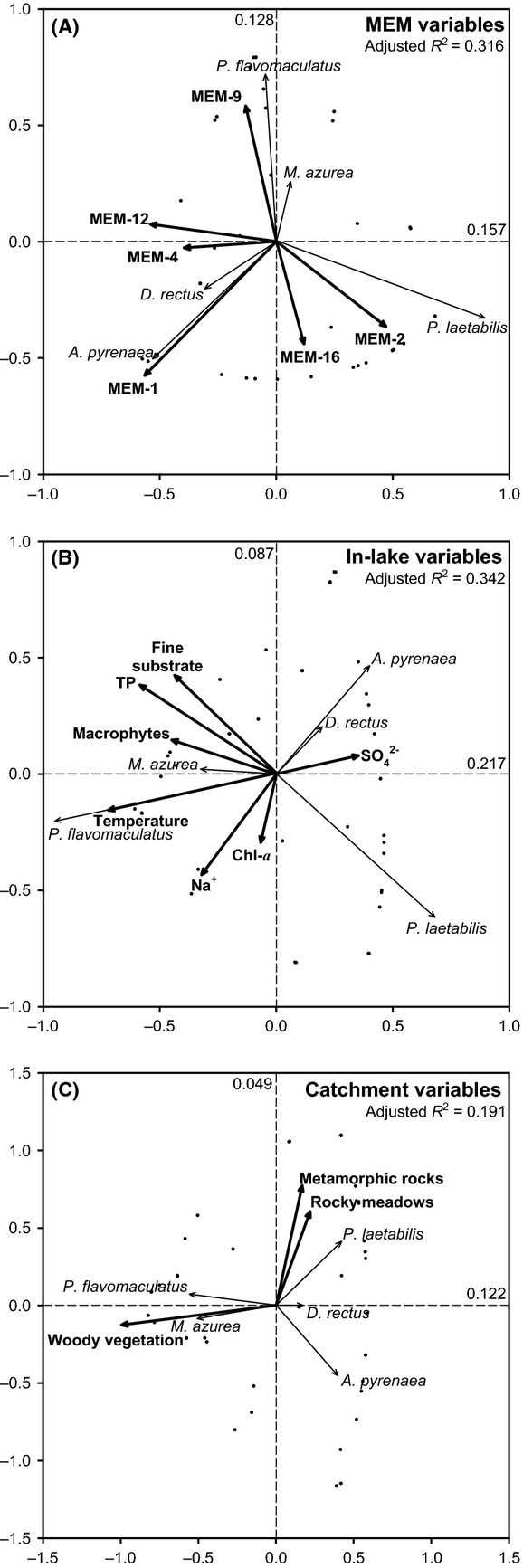
Biplots of redundancy analyses (RDAs) of the five most frequent Trichoptera species using (A) MEM variables, (B) in-lake environmental variables, and (C) catchment environmental variables. Adjusted *R*^2^ values are indicated for the overall analysis and for each of the two main axes in each plot. Scaling based on interspecies correlations. Table2 includes details on the forward selection of variables.

Six MEM variables were selected as significant for describing the spatial autocorrelation in the species assemblage composition (Fig.2), namely from broad scale to fine scale: MEM-1, MEM-2, MEM-4, MEM-9, MEM-12, and MEM-16. On a large scale, MEM-1, MEM-2, and MEM-4 indicated longitudinal patterns, correlating with the distribution of species with longitudinal bias in redundancy analysis (RDA) (Fig.3a): *P. flavomaculatus*, *M. azurea,* and *D. rectus* related to MEM-1, and *P. laetabilis* to MEM-2 and MEM-4. The widespread *A. pyrenaea* also followed MEM-1, as abundance values of this species declined toward the east (*n* = 60, *r* = −0.353 and *P* = 0.006, Appendix S1). Fine-scale MEM variables also contributed to explain species distributions, particularly MEM-9 in relation to *P. flavomaculatus*, MEM-12 to *P. laetabilis*, and MEM-16 to *M. azurea*. Overall, the species composition variance accounted for by MEM variables (adjusted *R*^2^ value) was 0.316.

### Environmental factors

The most relevant environmental factors in the in-lake and catchment RDAs were temperature and woody vegetation coverage, respectively, as indicated by forward selection of the variables (Table2). Species showing altitudinal bias segregated accordingly in the RDA (Fig.3b and c) along the first canonical axis: *M. azurea* and *P. flavomaculatus* were related to warmer lakes of higher productivity and in catchments more vegetated; whereas *A. pyrenaea* and *D. rectus* showed the opposite pattern, together with *P. laetabilis,* which showed no significant altitudinal bias. In fact, this latter species showed strong association for rocky environments both at in-lake and catchment analyses. The species composition variance accounted for by in-lake and catchment environmental variables (adjusted *R*^2^ values) was 0.342 and 0.191, respectively.

**Table 2:** Forward selection of variables in redundancy analysis (RDA) for MEM, in-lake, and catchment variables explaining species distributions. Biplot scores on canonical axes and the cumulative adjusted *R*^2^ value after the subsequent addition of variables are indicated. Inclusion of variables in each subset was performed following forward selection with Monte Carlo permutation tests (*P* < 0.05, 9999 permutations), where the double-stopping selection criterion of Blanchet et al. (2008) was applied

	adj *R*^2^	*P*	bs_1_	bs_2_
MEM variables explaining species distributions				
MEM-1	0.102	0.0003	−0.561	−0.576
MEM-2	0.159	0.0032	0.463	−0.365
MEM-9	0.205	0.0045	−0.129	0.583
MEM-12	0.252	0.0038	−0.535	0.073
MEM-4	0.285	0.0112	−0.394	−0.027
MEM-16	0.316	0.0144	0.114	−0.435
*Plectrocnemia laetabilis*	–	–	0.909	−0.341
*Polycentropus flavomaculatus*	–	–	−0.046	0.713
*Annitella pyrenaea*	–	–	−0.529	−0.503
*Drusus rectus*	–	–	−0.306	−0.202
*Mystacides azurea*	–	–	0.060	0.257
In–lake variables explaining species distributions				
Surface temperature	0.131	0.0001	–0.720	–0.160
TP	0.174	0.0059	–0.586	0.389
Na^+^	0.210	0.0042	–0.317	–0.434
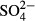	0.239	0.0162	0.351	0.078
Chl–*a*	0.289	0.0020	–0.064	–0.293
Macrophytes	0.310	0.0368	–0.447	0.142
Fine substrates	0.342	0.0085	–0.436	0.426
*Plectrocnemia laetabilis*	–	–	0.678	–0.612
*Polycentropus flavomaculatus*	–	–	–0.954	–0.207
*Annitella pyrenaea*	–	–	0.400	0.461
*Drusus rectus*	–	–	0.199	0.205
*Mystacides azurea*	–	–	–0.325	0.023
Catchment variables explaining species distributions				
Woody vegetation	0.131	0.0001	–0.990	–0.129
Metamorphic rocks	0.165	0.0121	0.170	0.771
Rocky meadows	0.191	0.0323	0.221	0.592
*Plectrocnemia laetabilis*	–	–	0.424	0.413
*Polycentropus flavomaculatus*	–	–	–0.556	0.070
*Annitella pyrenaea*	–	–	0.398	–0.443
*Drusus rectus*	–	–	0.173	–0.005
*Mystacides azurea*	–	–	–0.502	–0.082

adj *R*^2^, cumulative adjusted *R*^2^ values; bs_1_ and bs_2_, biplot scores with first and second axes.

### Variance partitioning

Variance partitioning (partial RDA) with MEM and environmental variables (Table3) revealed that the fraction of species assemblage variation that could be attributed uniquely to environment influence was about twofold larger than the variation uniquely attributable to the spatial structure. Although MEM variables accounted for a fraction of species composition variance comparable to that of in-lake variables and higher than that of catchment variables, most of its explanatory power was actually shared with the environmental variables. The variation explained by the longitudinal trend was low and completely shared with either environmental variables or MEM components (Table3).

**Table 3:** Variance partitioning (partial RDA) between spatial autocorrelation and environmental factors

	Adjusted *R*^2^ values	
	Total	Unshared
All variables	**0.501**	–
Environmental factors	**0.410**	**0.186**
In–lake variables	0.342	0.106
Catchment variables	0.191	0.047
Spatial autocorrelation	**0.315**	**0.091**
MEM variables	0.316	0.065
Linear longitudinal trend	0.072	–0.004

### Correlation between MEM and environmental variables

Broad-scale patterns of spatial autocorrelation (MEM-1, MEM-2, and MEM-4) correlated significantly (*P* < 0.05) with some environmental variables that indicate thermal conditions, general trophic status, or vegetation coverage of the catchments (Table4). This is in agreement with the RDA results (Fig.3), in which the distributions of *P. flavomaculatus* and *M. azurea* were positively related to temperature and woody vegetation, and negatively related to MEM-1 (contrary to *D. rectus* and *A. pyrenaea*). Total phosphorus and fine substrates were marginally correlated (*P* < 0.10) with MEM-4, in agreement with the inverse relationship between *P. laetabilis* and these two environmental variables (and MEM-4) in RDA.

**Table 4:** Pearson product–moment correlation coefficient *r* between environmental and MEM variables

	MEM–1	MEM–2	MEM–4	MEM–9	MEM–12	MEM–16
In–lake variables						
Surface temperature	**–0.384**	–0.148	0.202	0.169	–0.086	–0.121
TP	–0.082	**–0.365**	*0.218*	0.113	0.069	0.028
Na^+^	**–0.403**	–0.168	0.005	0.142	–0.166	–0.052
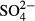	0.067	–0.132	–0.081	*–0.241*	*–0.222*	–0.038
Chl–*a*	**–0.386**	*0.220*	0.167	–0.029	–0.140	0.122
Macrophytes	–0.107	–0.037	0.078	0.036	0.141	*–0.219*
Fine substrate	–0.136	–0.168	*0.212*	0.036	0.025	–0.145
Catchment variables						
Woody vegetation	**–0.292**	–0.179	**0.306**	0.150	0.025	–0.173
Metamorphic rocks	–0.072	0.075	**–0.321**	–0.093	–0.063	–0.147
Rocky meadows	–0.020	0.191	–0.188	–0.084	–0.094	–0.003

Environmental variables within each subset are arranged following the order of selection in RDA (Table2). Significant correlations (*P* < 0.05) are highlighted in boldface; marginally significant correlations (*P* < 0.10) are shown in italics.

In contrast to large-scale patterns, the spatial autocorrelation at a fine scale (MEM-9, MEM-12, and MEM-16) was scarcely related to the environment (Table4). Yet, the relationships between temperature and MEM-9, and between macrophytes and MEM-12, were both significant when considering only lakes located at the western extreme (not shown in Table4, *n* = 12, *r* = 0.583, and *P* = 0.047 for temperature, *r* = −0.791 and *P* = 0.002 for macrophytes). These results also agree with the RDA: a positive relationship was found between *P. flavomaculatus* and MEM-9 (and temperature), and *D. rectus* and *A. pyrenaea* related positively to MEM-12 but negatively to macrophytes (Fig.3).

### The most relevant environmental factor for each species

**Figure 4:**
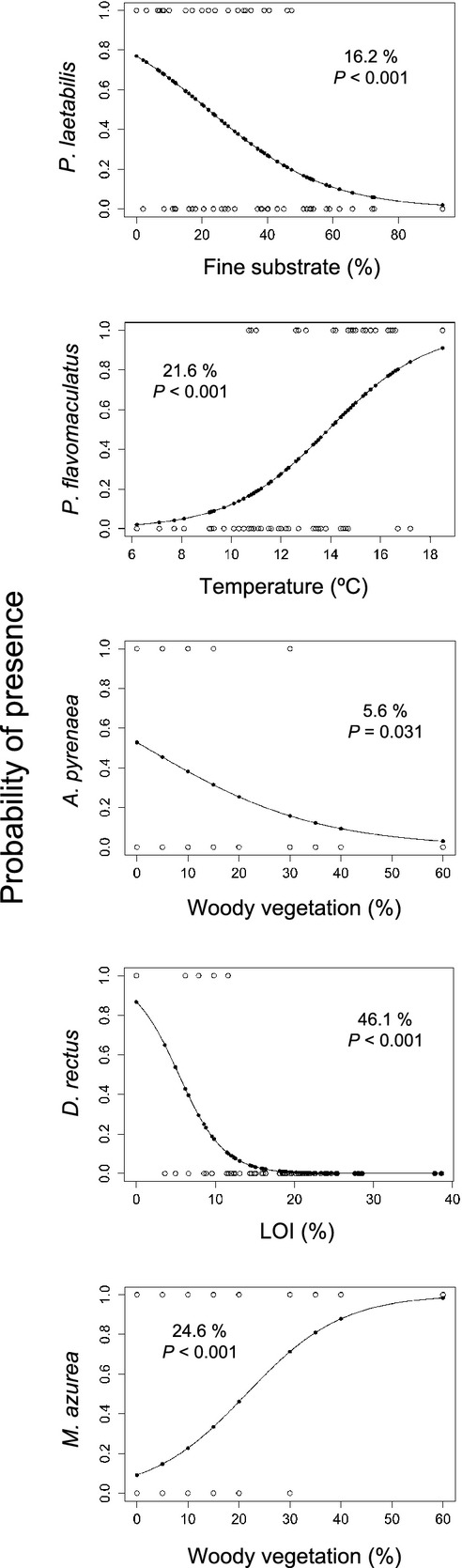
Probability of occurrence for each species as a function of the most explicative variable (lowest AIC) according to a generalized linear model (GLM, family = binomial, link = logit) using the same lakes as in previous RDA (*n* = 60). Percentage numbers inside each plot indicate the null deviance explained, with associated *P*-values (chi-square test on a deviance table). Information for all the variables in regard to AIC values and the null deviance explained (including its statistical significance) is available in Table5.

Binomial logistic GLMs revealed the variable most explicative of the geographical distribution of each species (Fig.4). For all species, the most relevant variable was one among those selected in the previous RDAs, with the sole exception of *D. rectus*. For this species, organic matter content in deep sediments (LOI) was selected. LOI can be considered a surrogate of lake general trophic status and is significantly correlated with temperature (*n* = 60, *r* = 0.523, *P* < 0.001) and MEM-1 (not shown in Table4, *n* = 60, *r* = −0.414, *P* = 0.001). The variables selected for the other species were fine substrates (negatively correlated with *P. laetabilis*), temperature (positively correlated with *P. flavomaculatus*), and woody vegetation in lake catchments (positively correlated with *M. azurea* and negatively correlated with *A. pyrenaea*).

In terms of AIC values, for three species (*P. laetabilis*, *D. rectus,* and *M. azurea*), no other model was within 2 AIC units from the lowest AIC value (Table5). For *P. flavomaculatus*, temperature and LOI yielded similar results, although these two variables are correlated (see above). In contrast, for *A. pyrenaea* six different models were within 2 AIC units. The lowest AIC value also implied the largest amount of null deviance explained among all variables considered (Table5). The null deviance explained was high for *D. rectus* (46.1%) and low for *A. pyrenaea* (5.6%), with intermediate values (15–25%) for the other three species.

**Table 5:** AIC values of generalized linear models (GLM, family = binomial, link = logit) for each species, with one environmental variable at a time, and percentage of null deviance explained (% Dev.). The lowest AIC values within 2 units are in boldface for each species

Variables	*Plectrocnemia laetabilis*		*Polycentropus flavomaculatus*		*Annitella pyrenaea*		*Drusus rectus*		*Mystacides azurea*	
	AIC	% Dev.	AIC	% Dev.	AIC	% Dev.	AIC	% Dev.	AIC	% Dev.
In–lake										
Lake area	85.32	0.22	84.29	0.58	86.25	0.39	41.67	3.44	64.04	0.00
Lake depth	80.57	6.07*	84.76	0.00	86.52	0.06	42.01	2.56	63.87	0.30
Surface temperature	80.76	5.82*	**67.34**	21.57***	84.83	2.11	29.87	33.67***	57.12	11.54**
pH	82.64	3.52	84.46	0.38	86.24	0.41	41.16	4.75	63.97	0.13
Conductivity	81.02	5.50*	81.90	3.54	86.21	0.44	42.77	0.60	63.20	1.42
Macrophytes	74.95	12.95**	79.80	6.14*	86.32	0.61	36.07	17.80**	64.05	0.00
Fine substrates	**72.30**	16.20***	83.77	1.23	86.57	0.01	41.09	4.92	62.00	3.42
Gravel	83.38	2.61	84.45	0.38	84.48	2.54	42.59	1.06	64.05	0.00
Stones	84.71	0.98	84.76	0.00	86.29	0.34	42.93	0.20	62.37	2.79
Rocks	78.73	8.30**	84.04	0.89	86.40	0.21	39.70	8.49	63.27	1.30
Si	84.68	1.01	84.49	0.33	85.12	1.76	39.79	8.24	62.39	2.76
DOC	74.90	13.01**	81.53	4.00	86.56	0.02	42.77	0.63	61.16	4.82
NH_4_^+^	78.56	8.52**	84.65	0.14	85.25	1.61	42.78	0.59	61.53	4.20
Ca^2+^	80.10	6.63*	83.00	2.19	86.47	0.13	42.70	0.78	63.33	1.19
Mg^2+^	85.18	0.40	83.22	1.91	86.25	0.40	42.69	0.81	62.11	3.22
Na^+^	85.21	0.36	82.77	2.47	**82.15**	5.37^*^	42.63	0.96	58.22	9.71*
K^+^	82.61	3.55	84.69	0.09	86.49	0.10	39.62	8.69	60.29	6.25
ANC	79.39	7.50*	84.51	0.31	86.53	0.05	42.94	0.18	64.02	0.04
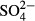	85.43	0.09	76.65	10.04**	85.47	1.34	42.98	0.07	56.93	11.86**
Cl^−^	80.71	5.89*	84.63	0.16	86.46	0.15	39.47	9.07	64.05	0.00
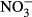	85.47	0.04	75.32	11.68**	86.10	0.57	28.44	37.34***	55.42	14.36**
Total nitrogen	79.29	7.62*	82.89	2.32	**83.31**	**3.96**	38.69	11.08*	60.63	5.69
Total phosphorus	81.45	4.97*	77.68	8.76**	85.03	1.88	42.94	0.19	58.31	9.56*
Chl–*a*	81.45	4.97*	84.19	0.70	86.50	0.10	36.64	16.33*	59.97	6.79*
Bacteria	75.64	12.11**	78.40	7.88*	**82.05**	5.48*	39.00	10.27*	57.52	10.88*
LOI in deep sediment	84.36	1.40	**68.42**	20.23^*^^*^^*^	85.13	1.75	**25.03**	46.08***	53.53	17.52**
Salmonidae	85.22	0.35	71.01	17.03***	84.75	2.21	38.73	10.98*	54.92	15.20**
*Phoxinus*	84.00	1.85	75.52	11.45**	**81.95**	5.60*	38.75	10.91*	57.95	10.16*
Catchment										
Catchment area	85.38	0.15	83.85	1.12	86.23	0.42	42.80	0.54	62.75	2.17
Woody vegetation	78.71	8.34**	81.38	4.19	**81.94**	5.61*	36.44	16.84*	**49.25**	24.64***
Peat bogs	85.37	0.17	83.49	1.58	85.64	1.13	41.91	2.83	64.01	0.06
Meadows	84.81	0.85	81.31	4.27	86.34	0.28	33.08	25.46**	64.05	0.00
Rocky meadows	77.92	9.30**	83.61	1.43	86.17	0.50	38.60	11.30*	63.80	0.41
Scree	84.88	0.76	78.48	7.78*	85.82	0.92	38.44	11.71*	60.64	5.68
Bare rocks	85.39	0.14	81.53	4.00	84.77	2.18	37.41	14.35*	62.50	2.58
Glacial deposits	83.30	2.71	82.67	2.59	**83.30**	**3.97**	39.46	9.10	63.14	1.51
Glaciers	83.30	2.71	82.67	2.59	86.29	0.35	38.98	10.32*	63.14	1.51
Metamorphic rocks	85.00	0.61	84.17	0.74	**83.17**	**4.13**	41.99	2.61	61.41	4.39
Plutonic rocks	85.29	0.26	81.65	3.85	86.58	0.00	42.59	1.08	56.61	12.39**
Detrital rocks	85.47	0.04	84.44	0.40	**82.90**	**4.45**	40.17	7.28	57.98	10.10*
Carbonate rocks	85.06	0.54	79.50	6.52*	85.02	1.89	42.36	1.65	62.10	3.24

DOC, dissolved organic carbon; ANC, acid neutralizing capacity; LOI, percentage of organic matter (loss on ignition). Asterisks indicate the significance of the explained deviance (chi–squared test on a deviance table):

* *P* < 0.05

** *P* < 0.01

*** *P* < 0.001.

## Discussion

### Environmental influences prevail over dispersal restrictions

Analyzing the relative influences of environmental and spatial factors on the assembly and distribution of aquatic insect species is essential for better understanding ecological communities in streams and lakes, with implications in conservation biology (Heino and Peckarsky 2014). It has been shown that the spatial extent considered affects the performance of models relating species assemblages and local environmental variables (Mykrä et al. 2007; Ilmonen et al. 2009; Heino 2011; Heino and Peckarsky 2014). At the spatial scale of the Pyrenees, our results indicate that environmental constraints, rather than dispersal limitations, prevail in the regional assembly and distribution of Trichoptera species in mountain lakes. This result differs from what was suggested in boreal lakes (Hoffsten 2004). The discrepancy between high-mountain and boreal lakes may arise from the smaller size of the mountain lake district and the stronger environmental changes at short-spatial scales due to altitude (e.g., temperature, vegetation, soils, lithology), or alternatively, from differences in dispersal ability of species between the two geographical contexts, which seems unlikely despite that our current knowledge on Trichoptera active aerial dispersal is limited.

Direct observations of Trichoptera flying adults indicate that aerial dispersal can persist over kilometric distances, although the capacity differs between species (Kovats et al. 1996) according to the respective flight morphology (Hoffsten 2004; Müller-Peddinghaus 2011; Müller-Peddinghaus and Hering 2013). However, it is unclear whether widespread species are also those that disperse the best. For example, the apparently low dispersal capacity of *P. flavomaculatus* does not preclude a widespread distribution of the species across Europe (Illies 1978), although there is a higher genetic differentiation of *P. flavomaculatus* among populations (Wilcock et al. 2007) than for species of higher dispersal capacity such as *Plectrocnemia conspersa* (Curtis), of the same family but with larger wings and body (Müller-Peddinghaus 2011). The relationship between the size of the distribution range of the species and their dispersal capacity requires more investigation as both features do not necessarily indicate the same. Mediterranean species often show high dispersal potential (Bonada et al. 2005), and boreal species show high variability in flight morphology and thus dispersal capacity (Hoffsten 2004). Endemic species are often regarded as weak dispersers (Hering et al. 2009; Previšić et al. 2014), but paradoxically, the only species found with widespread distribution at a Pyrenean scale is *A. pyrenaea* (Fig.1), the only one endemic to the Pyrenees among the species considered (Illies 1978). In summary, there is neither empirical evidence nor conceptual to sustain that the dispersal potential of species differs between mountain and boreal areas.

Spatial autocorrelation and environmental variables both explained a large fraction of species composition variance in this study (Fig.3, Table2). However, variance partitioning shows that the fraction of variance uniquely explained by environmental variables was more than twofold larger than that uniquely attributable to spatial autocorrelation (Table3). The overwhelming explicative capacity of the environment with respect over spatial autocorrelation indicates that dispersal constraints play a secondary role in the regional assembly and distribution of the most common Trichoptera species in the Pyrenean lakes. Furthermore, the geographically restricted distribution of some species (Fig.1, Table1) is explained by the patchy distribution of the environmental conditions. There is a high concordance between the explicative MEMs (Fig.2) and some environmental variables in the RDAs on species distributions (Fig.3, Table4), and the GLMs support the individual species–environment relationships indicated in the RDAs (Fig.4, Table5). Therefore, we can conclude that the presence of large environmental gradients related to altitude and landscape heterogeneity are of high significance in mountain areas and eventually prevail over dispersal constraints in explaining the Trichoptera species distributions, despite dispersal barriers across valleys.

### Species–environment relationships and the altitudinal distribution of Trichoptera

The altitudinal range of *D. rectus* observed in our study is narrow (only found above 2500 m a.s.l., Table1) but wide in nearby streams, where it reaches altitudes below 1500 m in the southern slope of the Pyrenees (Ventura 1998), and below 1000 m in the northern slope (Décamps 1967). *D. rectus* is a rheophilic species that attains high densities in cold and well-oxygenated waters in the Pyrenean streams (Décamps and Pujol 1975). Therefore, at the low edge of its altitudinal distribution, the species prefers fast currents (Décamps 1968), which are better oxygenated than slow flows. Our GLM results indicate that *D. rectus* respond negatively to organic matter content in deep sediments, a surrogate of lake general trophic status; correspondingly, the altitudinal tendency is clearly biased toward high elevations (median 2616 m in our data set), where lakes are less productive. We argue that *D. rectus* may surmount ventilation difficulties in lakes using cold waters, in which oxygen dissolves better and usually there is less consumption by organic matter decomposition. This explains the geographical pattern (i.e., altitudinal and longitudinal) observed for this species in our lake survey.

*M. azurea* is another example on how caddisfly species, even responding to similar proximal environmental restrictions, may show different altitudinal distributions when comparing lentic and lotic environments. Yet this species shows a negative altitudinal tendency in our study (Table1), it is frequently found at much lower altitudes in the streams of the Pyrenees (Décamps 1968; Cayrou et al. 2000) and nearby areas such as the Dordogne River catchment in southern France (Faessel 1985) and the rivers of the Mediterranean coast (Bonada et al. 2004). In streams, this species tends to inhabit in slow-current zones (e.g., Verneaux and Rezzouk 1971; Wallace et al. 1990), where both the terrestrial debris and fine organic matter sediment accumulate. *M. azurea* often feeds on macrophytes, yet not exclusively (Tachet et al. 2010), and builds soft cases with vegetal material, including pieces of terrestrial origin that provide consistency to the cases. In the mountains, the required microhabitats are hard to find in high-mountain streams, where the flow is too energetic, whereas they are more likely in low altitude lakes located in woody vegetation catchments, which is a general surrogate for availability of appropriate material for building the cases in the lakes. The geographically restricted distribution of *M. azurea* in the lakes of the Pyrenees probably simply mirrors the distribution of suitable habitats. Although woody vegetation in-lake catchments is the most relevant variable for both *M. azurea* and *A. pyrenaea*, the explained null deviance by this variable is very different between the two species (24.6% and 5.6%, respectively, Fig.4). This reflects that *M. azurea* has a strong dependence on vegetal material for case building, whereas the widespread *A. pyrenaea* may use both vegetal pieces and mineral grains, as observed in our samples. Accordingly, Feio et al. (2005) found a positive association between woody vegetation and *M. azurea* populations in the Mondego River basin (central Portugal).

In contrast with the previous species cases, the distribution of *P*. *laetabilis* and *P. flavomaculatus* agrees between lakes and streams. In our lake survey, their distribution is attributable to their different preferences for temperature and substrate type. Although *P. laetabilis* has been found in streams at relatively low altitudes (i.e., 650 m a.s.l.) in Galicia (northwestern Spain) (Vieira-Lanero et al. 2003), in the streams of the northern slope of the Pyrenees (France), *P. laetabilis* is commonly replaced by *P. conspersa* at low altitudes (Décamps 1968; Giudicelli et al. 1980; Cayrou et al. 2000), and in the southern (and warmer) half of the Iberian Peninsula *P. laetabilis* is rarely found, in contrast to *P. flavomaculatus* (González et al. 1992; Ruiz et al. 2001; Bonada et al. 2004). In the Pyrenees, *P. flavomaculatus* is much more frequent in the southern than in the northern slope, because in the latter it tends to appear at much lower altitudes, outside the range of mountain lakes (Décamps 1968; Giudicelli et al. 1980; Cayrou et al. 2000). Solem and Birks (2000) also noted the thermophily of *P. flavomaculatus* in the sediment record of Kråkenes Lake (western Norway), and Jacobsen and Brodersen (2008) showed that the oxy-regulatory capacity of the genus *Polycentropus* is greater at high than at low temperatures, in contrast to most other macroinvertebrate taxa analyzed, suggesting that oxygen depletion is not as constraining for *P. flavomaculatus* as for some other Trichoptera species. Concerning substrate type preferences, in a previous study on substrate preferences along a transect in Lake Redon (Pyrenees), *P. laetabilis* was found only in stony substrates (Rieradevall et al. 1999), in agreement with our results. In the subalpine lake Øvre Heimdalsvatn (southern Norway), *P. flavomaculatus* was dominant in stones too, but was not exclusively found in this type of habitat (Lillehammer 1978). For another *Polycentropus* species, *P. variegatus* Banks, a preference for gravel rather than bedrocks or silt was reported in stream channels in the Oregon Coast Range (Wevers and Wisseman 1987). Therefore, it could be possible that *Polycentropus* species are more prone than *Plectrocnemia* to survive in fine substrates, following the oxy-regulatory capacity of the genus (Jacobsen and Brodersen 2008).

### Beyond the Trichoptera case

A general conclusion from our study, beyond the particular case of Trichoptera, is that the spatial distribution of the environmental gradients (not only the overall strength of it) may be relevant as a counterpart of the influence of dispersal capacity in determining species distribution. This may produce a contrasting relative role of environment filtering between boreal and Pyrenean lakes in the Trichoptera distribution. On the other hand, the marked differences between lakes and streams in the altitudinal distribution of some Trichoptera species indicate that the proximal environment is the one that matter most. How some specific characteristics of the proximal environment distribute across the landscape (e.g., water oxygen availability) may differ substantially between lentic and lotic systems and, as a consequence, determine contrasting geographical (altitudinal in our case) distributions for populations of the same species in the two habitats, which may have consequences for the metapopulation dynamics.

## Supporting Information

**Appendix S1.** Taxonomy and detailed distribution of Trichoptera in the lakes studied. **Appendix S2.** Descriptive statistics of the environmental variables. **Appendix S3.** Estimation of the lake connectivity matrix for MEM analysis.
