# Supplementary material for: Environmental factors prevail over dispersal constraints in determining the distribution and assembly of Trichoptera species in mountain lakes
Source: Ecol Evol. 2015 Jun 1;5(13):2518–32. doi: 10.1002/ece3.1522 (PMC4523350; doi:10.1002/ece3.1522)
Supplement: Supplementary file 1 [file ece30005-2518-sd1.pdf]

Supporting Information to

Environmental factors prevail over dispersal constraints in determining the distribution and assembly of Trichoptera species in mountain lakes

Guillermo de Mendoza, Marc Ventura and Jordi Catalan

Ecology and Evolution

Appendix S1 Taxonomy and detailed distribution of Trichoptera in the lakes studied.

Appendix S2 Descriptive statistics of the environmental variables.

Appendix S3 Estimation of the lake connectivity matrix for MEM analysis.

Appendix S1 Taxonomy and detailed distribution of Trichoptera in the lakes studied.

For larvae determination, Tachet *et al.* (2010) was used as a starting reference, complemented with other general references of the Palearctic (Lepneva, 1964, 1966; Edington & Hildrew, 1981; Moretti, 1983; Wallace *et al.*, 1990; Camargo & García de Jalón, 1998). More specialised taxonomical studies were also used for Polycentropodidae (Edington, 1964; Wallace & Wallace, 1983; Vieira-Lanero *et al.*, 2003a,b), Limnephilidae (Décamps, 1961; Décamps & Pujol, 1975; Szczęsny, 1978; Higler & Solem, 1986; Sipahiler, 1998), Leptoceridae (MacDonald, 1950; Wallace, 1981), and Uenoidae (Giudicelli, 1971). Trichoptera pupae were also considered, and determined following Malicky (1983) when sufficiently mature.

Not all individuals could be determined to the species level. Hence, some words of caution are necessary concerning *Plectrocnemia*, *Annitella* and *Drusus* species assignments. Among *Plectrocnemia*, we always found *P. laetabilis* McLachlan when the determination was possible (10 lakes), but there were samples in which only larval individuals in an early stage of maturation were present, these cases are indicated in the table below. For *Annitella*, we only collected larval individuals, and all of them matched the description of Décamps (1961) for *A. pyrenaea* (Navás), a widespread species in the Pyrenees (Décamps, 1967a; Sipahiler, 1998) and, up to present, the only species of the genus ever cited in these lakes (Décamps, 1967a,b, 1968; Capblancq & Laville, 1983), although *A. obscurata* (McLachlan) has been cited in stream habitats in the central Pyrenees (Décamps, 1967b, 1968; Bautista, 1980). All the specimens of *Drusus* were also in a larval stage. They matched the description of *D. rectus* McLachlan larval stage found within the taxonomic references used. *D. marinettae* Sipahiler, with unknown larva, has been described from the eastern Pyrenees (Sipahiler, 1992).

Appendix S1

In the next table, the detailed distribution of the Trichoptera taxa found is shown for all those lakes where at least one individual has been collected (n = 63). Lakes are arranged by increasing longitude values. Abbreviations are as follows: Lat, Latitude N (º); Lon, Longitude E (º); Alt, Altitude (m a.s.l.); *Ple*, *Plectrocnemia*; *Pol*, *Polycentropus flavomaculatus* (Pictet); *Agr*, *Agrypnia*; *Dru*, *Drusus* cf. *rectus* McLachlan; *Lim*, *Limnephilus*; *Pot*, *Potamophylax latipennis* (Curtis); *Ann*, *Annitella* cf. *pyrenaea* (Navás); *Thr*, *Thremma gallicum* McLachlan; *Ath*, *Athripsodes aterrimus* (Stephens); *Mys*, *Mystacides azurea* (Linnaeus); ind, indeterminate (i.e. not determined to genus level). Asterisks indicate those samples in which either *Plectrocnemia* or *Limnephilus* individuals were determined to species level: *, *Plectrocnemia laetabilis* McLachlan determined; **, *Limnephilus guadarramicus* Schmid determined. Trichoptera numbers refer to as abundance of individuals found.

Appendix S1

Lake	Lat	Lon	Alt	*Ple*	*Pol*	*Agr*	*Dru*	*Lim*	*Pot*	*Ann*	*Thr*	*Ath*	*Mys*	ind
Acherito	42.881	-0.706	1875	0	2	0	0	0	1	0	0	0	0	0
Ormiélas	42.884	-0.356	1974	0	1	0	0	0	0	0	0	0	0	0
Asnos	42.692	-0.266	2060	0	0	1	0	0	0	0	0	0	0	0
Pondiellos Superior	42.777	-0.263	2745	0	0	0	0	0	0	0	0	0	0	1
Arnales	42.775	-0.242	2305	3	0	0	0	0	0	12	0	0	0	1
Arratille	42.802	-0.174	2247	0	0	0	0	2**	0	1	2	0	0	0
Estom	42.808	-0.099	1804	0	0	0	0	0	0	1	1	0	0	0
Glacé	42.778	-0.088	2571	0	0	0	4	0	0	0	0	0	0	0
Helado de Marboré	42.697	0.041	2592	0	0	0	0	0	0	1	0	0	0	0
Tourrat	42.810	0.100	2636	0	0	0	2	0	0	0	0	0	0	0
La Munia Superior	42.706	0.125	2537	0	0	0	8	0	0	0	0	0	0	0
Barroude Inferior	42.733	0.145	2377	0	0	0	0	0	0	2	0	0	0	0
Les Laquettes 1	42.836	0.148	2085	0	1	0	0	0	0	0	0	0	0	0
Port-Bielh	42.874	0.188	2290	0	45	0	0	0	0	0	0	0	0	0
La Mora	42.545	0.328	1908	0	0	0	0	0	0	21	0	0	0	0
Pixón	42.637	0.380	2199	0	3	0	0	0	0	3	0	0	0	0
Bachimala Superior	42.704	0.387	2630	0	0	0	2	0	0	0	0	0	0	0
Chelau Superior	42.624	0.407	2805	0	0	0	0	0	0	0	0	0	0	1
Posets	42.647	0.449	2550	0	0	0	0	0	0	7	0	0	0	0
Eriste	42.646	0.468	2411	0	0	0	0	0	0	84	0	0	0	0
Cregüeña	42.639	0.625	2640	0	0	0	1	0	0	29	0	0	0	0
Coronas	42.630	0.638	2740	0	0	0	9	0	0	15	0	0	0	1
Llosás	42.618	0.655	2480	17	0	0	0	0	0	6	0	0	0	0
Puis	42.655	0.708	2056	1	0	0	0	0	0	0	0	0	0	0
Redon	42.642	0.780	2235	18	5	0	0	0	0	0	0	0	0	0
Nere de Güerri	42.793	0.850	2280	14*	0	0	0	5	0	0	0	0	0	0
Pica Palomera	42.794	0.869	2308	0	0	0	0	12	0	4	0	0	0	0
Long de Liat	42.807	0.874	2140	0	7	0	0	0	0	1	0	0	0	0
Monges	42.623	0.877	2418	15	0	0	0	0	0	6	0	0	0	0
Montoliu	42.785	0.926	2375	1	0	0	0	0	0	1	0	0	0	0
Plan	42.622	0.931	2188	0	8	0	0	0	0	1	0	9	0	0
Llong	42.574	0.951	2000	1	0	0	0	0	0	0	0	0	3	0
Gelat de Bergús	42.591	0.963	2493	1	0	0	0	0	0	8	0	0	0	0
Illa	42.618	0.993	2452	4*	0	0	0	0	0	13	0	0	0	0
Gerber	42.631	0.995	2170	2	5	0	0	0	0	12	0	0	6	0
Airoto	42.703	1.039	2210	2	5	0	0	0	3	0	0	0	5	0
Gran de Mainera	42.525	1.046	2450	0	1	0	0	0	0	0	0	0	0	0
Rond	42.794	1.064	1929	17*	0	0	0	0	0	2	0	0	0	0
La Gallina Inferior	42.706	1.187	2270	45	0	0	0	0	0	3	0	0	0	0
Mariola	42.717	1.224	2276	95*	0	0	0	0	0	0	0	0	0	0
Senó	42.712	1.323	2130	0	15	0	0	0	0	0	0	0	1	0
Romedo de Dalt	42.706	1.325	2110	0	2	0	0	0	0	0	0	0	0	0
Aubé	42.745	1.338	2094	18	0	0	0	0	0	0	0	0	0	0
Aixeus	42.611	1.372	2370	30*	0	0	0	0	0	0	0	0	0	0
Sotllo	42.652	1.384	2346	12*	0	0	0	0	0	0	0	0	0	0
Angonella Superior	42.610	1.481	2440	9*	0	0	0	0	0	1	0	0	0	0
Tristaina Superior	42.647	1.487	2300	10*	1	0	0	0	0	6	0	0	0	0
Blaou	42.655	1.573	2350	8*	0	0	0	0	0	0	0	0	0	0
Gran de la Pera	42.458	1.595	2350	0	5	0	0	0	0	0	0	0	0	0
Ensangents Superior	42.521	1.649	2550	0	1	0	0	0	0	7	0	0	0	0
Montmalús	42.498	1.683	2440	2	7	0	0	0	1	0	0	0	1	0
Canals Roges	42.587	1.712	2410	1	0	0	0	0	0	2	0	0	0	0
Albe	42.618	1.745	2355	7*	0	0	0	0	0	2	1	0	0	0
Siscar	42.601	1.747	2187	0	4	0	0	0	0	0	0	0	0	0
Malniu	42.474	1.792	2250	0	13	0	0	0	0	0	0	0	56	0
Aygue Longue	42.642	1.883	2076	0	3	0	0	0	0	0	0	0	6	0
Trebens	42.578	1.963	2306	0	3	0	0	0	0	0	0	0	0	0
Blau	42.616	1.967	2531	7	2	0	0	0	0	0	0	0	0	0
Bleu de Rabassoles	42.700	1.973	1920	0	0	0	0	0	0	0	0	0	1	0
Gros de Camporrells	42.626	2.008	2255	0	36	0	0	0	0	0	0	0	7	0
Laurenti	42.675	2.026	1936	0	0	0	0	0	0	0	0	0	2	0
Gorg Negre	42.636	2.211	2083	0	10	0	0	0	0	0	0	0	2	0
Estelat	42.646	2.214	2021	0	0	0	0	0	0	2	0	0	5	0

Appendix S1

References

Bautista, M.-I. (1980) Contribució a l'estudi dels tricòpters d'Andorra. *Butlletí de la Institució Catalana d'Història Natural*, **45**, 89–96.

Camargo, J.A. & García de Jalón, D. (1988). Principales características morfológicas de los géneros ibéricos de la familia Limnephilidae (Trichoptera), en sus últimos estadios larvarios. *Boletín de la Asociación Española de Entomología*, **12**, 239–258.

Capblancq, J. & Laville, H. (1983) Le lac de Port-Bielh (Pyrénées): exemple de fonctionnement d'un écosystème lacustre de haute montagne. In: *Problèmes d'écologie. Structure et fonctionnement des écosystèmes limniques* (dir. by M. Lamotte & F. Bourlière), pp. 51–79. Masson, Paris.

Décamps, H. (1961). La larve et la nymphe de *Annitella pyrenaea* (Navas) (Trichoptère, Limnophilidae). *Bulletin de la Société Zoologique de France*, **86**, 763–770.

Décamps, H. (1967a) Écologie des trichoptères de la Vallée d'Aure (Hautes-Pyrénées). *Annales de Limnologie*, **3**, 399–577.

Décamps, H. (1967b) Introduction a l'étude écologique des trichoptères des Pyrénées. *Annales de Limnologie*, **3**, 101–176.

Décamps, H. (1968) Vicariances écologiques chez les trichoptères des Pyrénées. *Annales de Limnologie*, **4**, 1–50.

Décamps, H. & Pujol, J.-Y. (1975). Les larves de Drusinae des Pyrénées (Trichoptères, Limnephilidae). *Annales de Limnologie*, **11**, 157–167.

Edington, J.M. (1964). The taxonomy of British polycentropid larvae (Trichoptera). *Proceedings of the Zoological Society of London*, **143**, 281–300.

Edington, J.M. & Hildrew, A.G. (1981). *A key to the caseless caddis larvae of the British Isles with notes on their ecology*. Freshwater Biological Association, Ambleside.

Giudicelli, J. (1971). Monographie du genre *Thremma*. Trichoptera, Thremmatidae. *Annales de Limnologie*, **7**, 125–139.

Higler, L.W.G. & Solem, J.O. (1986). Key to the larvae of North-West European *Potamophylax* species (Trichoptera, Limnephilidae) with notes on their biology. *Aquatic Insects*, **8**, 159–169.

Lepneva, S.G. (1964). *Fauna of USSR. Trichoptera II (1). Larvae and pupae of Annulipalpia.* Israel Program for Scientific Translations (1970), Jerusalem.

Appendix S1

Lepneva, S.G. (1966). *Fauna of USSR. Trichoptera II (2). Larvae and pupae of Integripalpia*. Israel Program for Scientific Translations (1971), Jerusalem.

MacDonald, W.W. (1950). The larvae of *Mystacides azurea* L., *Cyrnus flavidus* McLachlan and *Oxyethira simplex* Ris (Trichoptera). *Proceedings of the Royal Entomological Society of London A*, **25**, 19–28.

Malicky, H. (1983). *Atlas of European Trichoptera*. Dr. W. Junk, The Hague.

Moretti, G. (1983). *Tricotteri (Trichoptera). Guide per il riconoscimento delle specie animali delle acque interne italiane* (coord. by S. Ruffo). Consiglio Nazionale delle Ricerche, Verona.

Sipahiler, F. (1992) Four new species of Drusinae from Spain and France (Insecta, Trichoptera, Limnephilidae). *Spixiana*, **15**, 285–291.

Sipahiler, F. (1998). Studies on the genus *Annitella* Klapálek (Trichoptera: Limnephilidae: Chaetopterygini) in the Iberian Peninsula. *Aquatic Insects*, **20**, 149–164.

Szczęsny, B. (1978). Larvae of the family Drusinae (Insecta: Trichoptera) from the Polish part of the Carpathian Mts. *Acta Hydrobiologica*, **20**, 35–53.

Tachet, H., Richoux, P., Bournaud, M. & Usseglio-Polatera, P. (2010). *Invertébrés d'eau douce: systématique, biologie, écologie (nouvelle édition revue et augmentée)*. CNRS, Paris.

Vieira-Lanero, R., González, M.A. & Cobo, F. (2003a). The larva of *Plectrocnemia laetabilis* McLachlan, 1880 (Trichoptera; Polycentropodidae; Polycentropodinae). *Annales de Limnologie – International Journal of Limnology*, **39**, 135–139.

Vieira-Lanero, R., González, M.A. & Cobo, F. (2003b). The larva of *Polycentropus corniger* McLachlan, 1884 and *Polycentropus intricatus* Morton, 1910 (Insecta, Trichoptera, Polycentropodidae, Polycentropodinae). *Spixiana*, **26**, 243–247.

Wallace, I.D. (1981). A key to the larvae of the family Leptoceridae (Trichoptera) in Great Britain and Ireland. *Freshwater Biology*, **11**, 273–297.

Wallace, I.D. & Wallace, B. (1983). A revised key to larvae of the genus *Plectrocnemia* (Polycentropodidae: Trichoptera) in Britain, with notes on *Plectrocnemia brevis* McLachlan. *Freshwater Biology*, **13**, 83–87.

Wallace, I.D., Wallace, B. & Philipson, G.N. (1990). *A key to the case-bearing caddis larvae of Britain and Ireland*. Freshwater Biological Association, Ambleside.

Appendix S2 Descriptive statistics of environmental variables (82 lakes). Abbreviations: sd, standard deviation; min., minimum value; max., maximum value; * mean for Salmonidae and *Phoxinus* is referred to as the frequency of occurrence.

	**mean**	**median**	**sd**	**min.**	**max.**
In-lake variables					
Lake area (ha)	8.05	5.58	8.78	0.24	53.19
Lake depth (m)	22.1	16.7	20.6	0.7	123.0
Surface temperature (ºC)	12.4	12.7	3.2	3.8	18.5
pH	7.10	7.00	0.79	4.53	8.96
Conductivity at 20 ºC (µS cm^{-1})	40.0	23.8	38.4	5.2	190.8
Macrophyte coverage (%)	9.1	0.0	19.0	0.0	100
Fine substrate coverage (%)	29.7	26.5	22.5	0.0	93.5
Gravel coverage (%)	8.8	6.3	8.4	0.0	32.5
Stone coverage (%)	19.2	18.8	10.8	0.0	46.7
Rock coverage (%)	42.3	40.7	20.3	0.0	83.0
Si (mg·Si l^{-1})	0.91	0.67	0.79	0.02	5.03
Dissolved organic carbon (mg C l^{-1})	1.06	0.78	1.23	0.00	9.42
NH_4^+ (µeq·l^{-1})	1.26	1.19	0.60	0.18	3.89
Ca^{2+} (µeq·l^{-1})	280.5	133.9	287.5	20.0	1194.6
Mg^{2+} (µeq·l^{-1})	43.8	15.1	99.2	4.0	557.3
Na^+ (µeq·l^{-1})	24.25	20.43	13.84	0.28	75.76
K^+ (µeq·l^{-1})	3.89	3.29	3.90	0.00	21.91
Acid neutralising capacity (µeq·l^{-1})	257.1	122.5	320.5	-40.0	1696.0
SO_4^{2-} (µeq·l^{-1})	92.85	41.29	184.55	10.27	1239.96
Cl^- (µeq·l^{-1})	8.08	6.72	4.77	3.29	36.61
NO_3^- (µeq·l^{-1})	6.30	5.19	5.41	0.00	19.90
Total nitrogen (µg·N l^{-1})	225.1	177.7	173.6	43.9	967.9
Total phosphorus (µg·P l^{-1})	4.63	3.36	4.55	0.94	33.27
Chl-*a* (µg Chl l^{-1})	2.10	1.26	2.88	0.03	19.07
Bacteria (µg C ^{1})	22.0	15.8	19.5	1.0	93.3
LOI in deep sediment (%)	16.4	17.2	8.8	0.0	40.9
Salmonidae (presence / absence) *	0.68	-	-	-	-
Phoxinus (presence / absence) *	0.32	-	-	-	-
Catchment variables					
Catchment area (ha)	246.9	113.1	622.3	7.0	5437.9
Woody vegetation coverage (%)	6.2	0.0	13.0	0.0	70.0
Peat bog coverage (%)	0.5	0.0	1.7	0.0	10.0
Meadow coverage (%)	20.9	15.0	23.4	0.0	90.0
Rocky meadow coverage (%)	10.5	0.0	16.3	0.0	78.0
Scree coverage (%)	29.3	25.0	22.9	0.0	100
Bare rock coverage (%)	32.0	30.0	20.2	0.0	90.0
Glacial deposits coverage (%)	0.3	0.0	1.6	0.0	10.0
Glacier coverage (%)	0.2	0.0	1.7	0.0	15.0
Metamorphic rock coverage (%)	26.1	0.0	38.5	0.0	100
Plutonic rock coverage (%)	47.7	40.0	46.7	0.0	100
Detrital rock coverage (%)	13.2	0.0	28.2	0.0	100
Carbonate rock coverage (%)	11.5	0.0	21.9	0.0	90.0

Appendix S3 Estimation of the lake connectivity matrix for Moran's Eigenvector Maps (MEM) analysis. The most likely matrix for lake connectivity was obtained by comparing the AICc values of a number of possible matrices, based on the MEM analysis of species assemblages. The procedure was performed in five steps.

Step 1 Several geometric connectivity schemes were first used to build binary (i.e. unweighted by distance) models of lake connectivity, following Dray *et al.* (2006), and using the packages 'SoDA' (Chambers, 2013) and 'spacemakeR' (Dray, 2013), available in R software (R Core Team, 2013). These connectivity matrices were, from simplest to most complex, the "minimum spanning tree", the "relative neighbourhood graph", the "Gabriel graph" and the "Delaunay triangulation", with the additional inclusion of the "distance-based criterion" (Dray *et al.*, 2006; Borcard *et al.*, 2011).

Step 2 Unlike other connectivity matrices, the distance-based criterion requires a prior definition of a *threshold distance* beyond which lakes are considered as unconnected. This distance can be obtained through a multivariate variogram, and equals to the largest distance at which the variogram is significant, considering only the lower half of the distance range (Wagner *et al.*, 2005; Dray *et al.*, 2006). In our case, this threshold distance was equal to the largest distance between lakes (120.7 km) that was within the lower half of the distance range. The empirical multivariate variogram of our caddisfly community data is represented in next page (95% confidence intervals shown as short-dash lines).

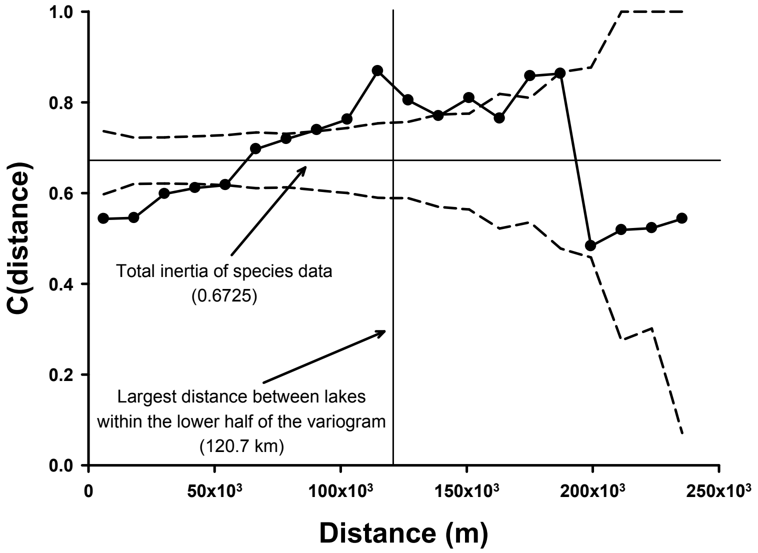

Step 3 The most adequate distance-based connectivity matrix must be one among all those possible distance matrices that can be built between a minimum distance, at which no lake is left unconnected (i.e. the "minimum spanning tree"), and the threshold distance above defined, beyond which lakes are considered too far apart to be connected. In our case, these two distance limits were 28.8 km and 120.7 km. Any distance-based matrix was considered as a potential candidate matrix if the *maximum distance between lakes*, hereinafter '*dmax*', was between these two limits.

Step 4 The number of matrices to be considered as candidates was further increased within each of the geometric matrix types defined in 'Step 1', by considering (or no) the addition of weights to links between lakes (i and j), as a decreasing function of their distance (d_{ij}). Following Dray *et al.* (2006), we considered three ways to define decreasing weights with distance: a linear function (f1), a concave-down function (f2) and a concave-up function (f3). This latter was here adapted to the geographical scale of our study (240 km), as only 4 m were considered as the threshold distance in Dray *et al.*

(2006), and the f3 suggested in that paper is strongly sensitive to scaling. The models here used for the connectivity matrices are summarised as follows:

Type of link	***Weighing function***	***Parameters ****	**Reference**
Binary	None	-	-
f1	$1 - d_{ij} / dmax$	*dmax*	Dray *et al*. (2006)
f2	$1 - (d_{ij} / dmax)^{a}$	*dmax* and *a*	Dray *et al*. (2006)
f3	$1 / (1 + d_{ij}^{1+z/10} / 4\ dmax\ d_{ij}^{1/dmax})$	*dmax* and *z*	this study

* *dmax* is to be estimated only for the 'distance-based' connectivity matrix, as it is fixed in other matrices; *a* and *z* are defined as integers and the values considered are from 1 to 10 (*a*) and from 1 to 5 (*z*)

Step 5 Following Dray *et al.* (2006), within each connectivity matrix and weighing function, MEM variables were used upon species assemblages, using Redundancy Analysis (RDA) with the R package 'vegan' (Oksanen *et al*., 2013) and after transformation of raw species data to obtain the Hellinger distance during RDA (Legendre & Gallagher, 2001). MEM variables where selected following forward selection and 9999 Monte Carlo permutations with the R package 'packfor' (Dray *et al*., 2013), by applying the double-stopping criterion of Blanchet *et al.* (2008). Only positive MEM, representing positive spatial autocorrelation, where considered for inclusion, as negative MEM are often the result of biotic interaction or "accidental" data structures (Borcard *et al*., 2011), which are out of the scope of the present study.

Final output

Following Dray *et al.* (2006), the best model (in terms of AICc) within each connectivity matrix and weighing function is shown below. The most likely connectivity matrix is the one with the lowest AICc value overall (shown in boldface).

Appendix S3

Connectivity matrix	Weighing function	# MEM variables	Adjusted R^2	AICc	Parameters
Minimum spanning tree	None	8	0.253	-29.4	
	f1	5	0.150	-26.3	
	f2	6	0.215	-29.6	$a = 7$
	f3	10	0.332	-32.7	$z = 1$
Relative neighbourhood	None	8	0.268	-30.7	
	f1	7	0.188	-26.0	
	f2	7	0.273	-32.7	$a = 5$
	f3	9	0.323	-33.7	$z = 4$
Gabriel graph	None	9	0.315	-32.9	
	f1	6	0.218	-29.8	
	f2	5	0.273	-35.6	$a = 7$
	f3	9	0.295	-31.2	$z = 5$
Delaunay triangulation	None	5	0.229	-32.2	
	f1	7	0.260	-31.6	
	f2	6	0.250	-32.4	$a = 6$
	f3	9	0.314	-32.9	$z = 2$
Distance-based	None	6	0.316	**-37.9**	*dmax* = 59.0 km
	f1	3	0.242	-35.9	*dmax* = 56.2 km
	f2	5	0.271	-35.5	*dmax* = 38.1 km; $a = 8$
	f3	10	0.339	-33.3	*dmax* = 66.6 km; $z = 5$

References

Blanchet, F.G., Legendre, P. & Borcard, D. (2008) Forward selection of explanatory variables. *Ecology*, **89**, 2623–2632.

Borcard, D., Gillet, F. & Legendre, P. (2011) *Numerical ecology with R*. Springer, New York.

Chambers, J.M. (2013) *SoDA: Functions and Examples for "Software for Data Analysis"*. R package version 1.0-6. http://CRAN.R-project.org/package=SoDA

Dray, S. (2013) *spacemakeR: Spatial modelling*. R package version 0.0-5/r113. http://R-Forge.R-project.org/projects/sedar/

Dray, S., Legendre, P. & Peres-Neto, P.R. (2006) Spatial modelling: a comprehensive framework for principal coordinate analysis of neighbour matrices (PCNM). *Ecological Modelling*, **196**, 483–493.

Dray, S., Legendre, P. & Blanchet, G. (2013) *packfor: Forward Selection with permutation (Canoco p.46)*. R package version 0.0-8/r109. http://R-Forge.R-project.org/projects/sedar/

Legendre, P. & Gallagher, E.D. (2001) Ecologically meaningful transformations for ordination of species data. *Oecologia*, **129**, 271–280.

Appendix S3

Oksanen, J., Blanchet, F.G., Kindt, R., Legendre, P., Minchin, P.R., O'Hara, R.B., Simpson, G.L., Solymos, P., Stevens, M.H.H. & Wagner, H. (2013). *vegan: Community Ecology Package*. R package version 2.0-9. http://CRAN.R-project.org/package=vegan

R Core Team (2013) *R: A language and environment for statistical computing*. R Foundation for Statistical Computing, Vienna. URL http://www.R-project.org/.

Wagner, H.H., Holderegger, R., Werth, S., Gugerli, F., Hoebee, S.E. & Scheidegger, C. (2005) Variogram analysis of the spatial genetic structure of continuous populations using multilocus microsatellite data. *Genetics*, **169**, 1739–1752.
